# Non-Commercial Grapevines Hybrids Fruits as a Novel Food of High Antioxidant Activity

**DOI:** 10.3390/foods11152216

**Published:** 2022-07-26

**Authors:** Remigiusz Olędzki, Krzysztof Lutosławski, Paulina Nowicka, Aneta Wojdyło, Joanna Harasym

**Affiliations:** 1 Department of Biotechnology and Food Analysis, Wrocław University of Economics and Business, Komandorska 118/120, 53-345 Wrocław, Poland; remigiusz.oledzki@ue.wroc.pl; 2 Adaptive Food Systems Accelerator-Science Centre, Wrocław University of Economics and Business, Komandorska 118/120, 53-345 Wrocław, Poland; 3 Department of Process Management, Wrocław University of Economics and Business, Komandorska 118/120, 53-345 Wrocław, Poland; krzysztof.lutoslawski@ue.wroc.pl; 4 Department of Fruit, Vegetable and Nutraceutical Plant Technology, Wrocław University of Environmental and Life Sciences, 37 Chełmońskiego Street, 51-630 Wrocław, Poland; paulina.nowicka@upwr.edu.pl (P.N.); aneta.wojdylo@upwr.edu.pl (A.W.)

**Keywords:** grape hybrids, antioxidants, polyphenols, reducing sugars, *Vitis labrusca*

## Abstract

Non-commercial hybrid grapevine cultivars, usually used for dessert purposes or as ornamental garden plants, may contain a wealth of bioactive substances and thus can be regarded as highly valuable food resources. Antioxidant properties and selected groups of polyphenolic components in the three fractions of fruits: peel, pulp and juice; of five hybrid grape cultivars grown in Poland—Michigan, Alwood, Minnesota, V68021 and Beta—were analyzed and characterized. The liquid chromatography coupled with tandem mass spectrometry (LC-MS-PDA-Q/TOF and UPLC-PDA), total polyphenols, flavonoids and anthocyanins, and DPPH, ABTS and FRAP were used for evaluation of antioxidant potential qualitatively and quantitatively as well as simple reductive sugars were measured. The antioxidant activity and polyphenols content depend mainly on the grape fruit fraction, while they depend to a lesser extent on the cultivar of the hybrid grapes studied. It was confirmed that grape skins are characterized by high antioxidant activity and their bioactive characteristics are similar to many hybrid grape cultivars grown in southern and Mediterranean regions of Europe. Especially grape skins of Alwood and Beta cultivars were characterized by a particularly high content of polyphenolic compounds, mainly from the flavonoid and anthocyanin group and a low content of simple sugars.

## 1. Introduction

The phenomenon of natural or planned generation of interspecific hybrids results in new secondary metabolites and increased activity of existing biosynthesis pathways. Studies conducted on the fruits of interspecific hybrids showed that, regarding the total content of phenolic compounds and their distribution in individual plant organs, hybrids can show enhanced traits inherited from both parental species [1].

The fruits of the vast majority of grapevine (Vitis) species are one of the richest sources of phenolic compounds belonging to such groups as anthocyanins, flavonols, tannins, phenolic acids and stilbenoids [2]. Studies conducted so far proved that the phytochemical and organoleptic characteristics of interspecific hybrids may be more favorable compared to the parent species, whose fruits usually have too high a content of simple sugars. For this reason, *Vitis vinifera* fruits in fresh or as juices should not be a part of a balanced diet, e.g., for diabetics who need to avoid products with a high glycemic index (GI ≥ 70). On the other hand, sometimes hybrid vine grapes, especially American grape varieties from *Vitis labrusca* species, are characterized by a strong aftertaste (described as “earthy”), which is unacceptable to consumers and excludes this fruit both from dessert applications and industrial uses. Despite the unfavorable sensory properties, studies showed that some American grapes (*V. labrusca*) and their hybrids with *Vitis vinifera*, may contain a significantly higher amount of bioactive compounds than products obtained from noble vine grapes [3].

Current results of physicochemical and biochemical studies revealed that stilbenoids, particularly abundant in Vitaceae spp., exhibit a wide range of biological health-promoting effects related to the prevention of obesity and related diseases such as coronary heart disease (ischemic heart disease), atherosclerosis or hypertension [4,5,6]. There is a known link between the consumption of *V. vinifera* fruit and a reduction of cancer occurrence, as it has been confirmed that the edible parts of grapevine (*V. vinifera*) fruit show the ability to inhibit the activity of aromatase and the nuclear transcription factor NFκB, which are currently seen as major factors in the early stages of cancer development, e.g., skin, mucous membranes and the uveal membrane of the eye, i.e., malignant melanoma [7].

In this study, we adopted the research hypothesis that the fruits of *Vitis* interspecific hybrids are characterized by a profile of bioactive substances, expressed in terms of polyphenol content and antioxidant and oxidoreductive activity that is more favorable compared to the fruits of the parent *Vitis* species. The available scientific literature lacks reports on the bioactive properties of dessert fruits from interspecific hybrids resulting from cross-breeding between *Vitis vinifera* and the American grape varieties *Vitis rupestris* and *Vitis labrusca*.

There is a paucity of studies that can provide knowledge on the bioactivity of grapevine fruit characteristics of Polish dessert and industrial varieties that are interspecific hybrids of *V. vinifera* with *V. labrusca* and *V. vinifera* with *V.rupestris* L. (rocky vine) or with *V. aestivalis* Mich × (summer grapevine). Therefore, the main objective of this study was to evaluate the content of bioactive substances with health-promoting, especially antioxidative effects, which is of particular importance for consumers of plant-origin functional foods. The antioxidant activity and the content of different groups of polyphenolic compounds in the fruits of five selected cultivars (Michigan, Minnesota, V68021, Beta and Alwood) of hybrid grapevines grown in Poland in the Rzeszow Upland (Lower San Valley) were evaluated.

## 2. Materials and Methods

### 2.1. Reagents and Chemicals

The standards of flavan-3-ols ((−)-epicatechin, (+)-catechin, procyanidins A2 and B2), and flavonols (quercetin-3-O-glucoside, isorhamnetin-3-O-rutinoside) used in identification of bioactive compounds in grapevine hybrids fruit were purchased from Extrasynthese (Lyon Nord, France). Chlorogenic, caffeic, and gallic acids, and also dephinidin-3-O-rutinoside, malvidin-3-O-glucoside and peonidin-3-O-glucoside were supplied by TRANS MIT GmbH (Giessen, Germany). All solvents for LC/MS grade (acetonitrile, and metanol) ewere purchased from Sigma-Aldrich (St. Louis, MO, USA). The following standards and reagents were used for the spectrophotometric methods: delphinidin 3-O-glucoside chloride (Sigma-Aldrich, St. Louis, MO, USA), rutoside (quercetin 3-rutinoside), glucose, fructose, Trolox (6-hydroxyl-2,5,7,8–tetramethylchromo-2-carboxylic acid), 2,2-diphenyl–1-picrylhydrazyl (DPPH), 2,2-azino-bis (3-ethyl benzothiazoline-6-sulphonic acid) (ABTS), TPTZ (2,3,5-triphenyltetrazolium chloride), gallic acid and iron (III) sulphate hydrate (Pol-Aura, Zabrze, Poland).

### 2.2. Hybrid Vine Varieties and Environmental Conditions of Cultivation Environmental Conditions

The hybrid grape varieties studied were grown in the area of Widna Góra near Jarosław, Poland (latitude: 49.9949535971, longitude: 22.6679244932, altitude 237 m). According to the Köppen classification, in Poland, where the site of cultivation of the hybrid vines studied is located, there is a humid continental climate zone, which in the climate classification system is labeled Dfb. Fruits were harvested in the morning at a stage of ripeness characterized by the skin coloring characteristic of each variety (Table 1).

The varieties tested were Michigan (American–European hybrid variety, green–yellow, general variety for dessert and for processing, e.g., fermentation); Minnesota (American hybrid, pink–violet, dessert variety); V68021 (American hybrid (Canada) Festivee × Illinois, light pink, dessert variety); Beta (American hybrid *Vitis riparia* × *Vitis labrusca*, dark blue, dessert variety arboricultural); Alwood (*Vitis vinifera* × *Vitis labrusca* navy–black or dark purple, commercial variety or dessert variety).

### 2.3. Preparation of Extracts for Analysis

Grape juice was obtained from fresh fruit. The grapes were washed thoroughly under running water and then drained. The grape fruits were crushed with a pestle (using a ceramic mortar) and the freshly pressed juice was separated using an acrylic fiber filter. Afterwards samples were frozen at −23 °C until analysis, and just before the analysis, the juice was centrifuged at 5000 rpm for 15 min (MPW-350, Warszawa, Poland).

The pulp was obtained by peeling the grape, removing the seeds and draining on blotting paper. 5 g of fresh raw material was weighed and macerated in a mixture (10 mL) of four solvents (methanol, acetone, water, acetic acid) mixed in proportions (40/40/18.5/1.5) and extracted for 12 h at room temperature without light. Then, the resulting extracts were centrifuged at 5000 rpm for 15 min (MPW-350, Poland) and the supernatant obtained was frozen at −23 °C until analysis. The skins were manually removed from the fruits and dried (in a dryer with natural air circulation). 5 g of dried skins were crushed using a mortar and infused into 10 mL of a mixture of four solvents (methanol, acetone, water, glacial acetic acid) mixed in proportions (40/40/18.5/1.5) for 12 h at room temperature in the absence of light [8,9]. Then, the extracts obtained were centrifuged at 5000 rpm for 15 min (MPW-350, Poland) and the supernatant obtained was frozen at −23°C until analysis. All the samples were analyzed in quadruplicate.

### 2.4. Determination of Total Phenolic Compounds

The total phenolic compound content was determined using the Folin–Ciocalteu reagent according to the method of Yen et al. (1995) [10], to which minor modifications were made. 0.1 mL of Folin–Ciocalteu reagent and 1.58 mL of H_2_O were added to the obtained extracts (0.02 mL). After 5 min of incubation, 0.3 mL of saturated sodium carbonate solution (Na_2_CO_3_) was added. The total phenolic compounds were determined after 20 min of incubation at 38 °C in the dark. The absorbance of the resulting solution was measured at 765 nm. A standard curve was prepared for gallic acid. TPC results were presented in milligrams of gallic acid equivalent (GAE) per 1 g of raw material used.

### 2.5. Determination of Antioxidant and Oxidoreductive Activities

#### 2.5.1. DPPH Test

The antioxidant capacity (against the 2,2-diphenyl-1-picrylhydrazyl radical) of the tested extracts and juice was measured according to the Klymenko method, with minor modifications [11]. 0.035 mL of the test solution was measured and added to 1 mL of (0.1 mM) methanolic DPPH solution. The mixture was shaken and left at room temperature for 20 min, after which the absorbance was measured at 517 nm. The antiradical activity was calculated from the calibration curve and expressed as mg Trolox equivalent (TE) for 1 g of raw material used.

#### 2.5.2. ABTS Test

The antiradical capacity against the cation radical of 2,2-azo-bis (3-ethyl benzothiazoline-6-sulfonic acid (ABTS-^+^) for the tested extracts and juice was measured based on the Sridhar method, with minor modifications [12]. ABTS-^+^ solution was prepared by mixing 7 mM ABTS stock solution with 2.45 mM potassium persulfate solution and incubating at room temperature (23 °C) in the dark for 16–24 h. The ABTS-^+^ solution was diluted with phosphate buffer (0.1 M) to give an absorbance of 0.700 ± 0.05 at 734 nm. To 1.0 mL of the diluted ABTS-^+^ solution, 0.02 mL of the plant extract or juice to be tested was added. The absorbance at 734 nm was read exactly 10 s after mixing the test extract with the ABTS-^+^ solution. The antiradical activity was calculated from the calibration curve and expressed as mg TE for 1 g of raw material used.

#### 2.5.3. FRAP Test

The reducing power (ability to reduce ferric ions) was measured according to the Re method, with minor modifications [13]. The test extract was added to 1 mL of FRAP solution (acetate buffer (300 µM, pH 3.6), a solution of 10 µM TPTZ in 40 µM HCl and 20 µM FeCl_3_ in the ratio 10:1:1 *v*/*v*). The mixture was shaken and left at room temperature for 20 min, after which the absorbance was measured at 593 nm. The reducing activity was calculated from the calibration curve and expressed as mg iron (II) sulfate equivalent FeSO_4_^2−^·7H_2_O for 1 g of raw material used.

### 2.6. Measurement of Total Flavonoid Content

Total flavonoid content in the samples tested (skins, pulp and juice) was determined using the aluminum chloride method according to the modified Chang’s method (to which minor modifications were made) using quercetin as a standard [14]. To 1 mL of the test sample was added 4 mL of water, 0.3 mL of 5% sodium nitrite, and the resulting mixture was incubated for 5 min at room temperature. Then 0.3 mL of 10% aluminum chloride was added and the contents were shaken. After 6 min of incubation at room temperature, 1 mL of 1 M sodium hydroxide was added to the mixture and incubated again for 15 min. The absorbance of the sample was measured spectrophotometrically at 510 nm. The results obtained were expressed as mg quercetin equivalent/g of the test material or as mg quercetin/mL of the test solution.

### 2.7. Measurement of Total Anthocyanin Content

For the determination of total anthocyanin content in skins, pulp and juice, the modified differential pH method according to Giusti was used, which is applicable to the determination of monomeric anthocyanins, expressed in fruit as delphinidin chloride. The determination of total monomeric anthocyanins is based on structural changes in the anthocyanin chromophore in an environment of pH 1.0–4.5 [15]. 10 mg of skin macerate or homogenate of fresh fruit pulp was weighed into a glass test tube, and 5 mL of a mixture of 1.5 M hydrochloric acid (HCl) and 5 mL of 95% methanol was added. The resulting solution was stirred, incubated for 10 min, centrifuged for 15 min (at 5000 rpm) and then the absorbance was measured at 535 nm. In the case of juice, 1 mL of diluted (1:2) juice was introduced into the tube, then a solution of 0.1 M HCl was added in an amount that reduced the pH of the solution to 3.5. The test solution was thoroughly mixed and allowed to stand for 5 min, after which the absorbance at 535 nm was measured. The total anthocyanin content was expressed as mg of delphinidin chloride equivalent per g of test material or ml of test juice.

### 2.8. Measurement of Reducing Sugar Content

The sugar content of grape juice was measured using a modified method according to Miller based on the reducing properties of sugars towards 3,5-dinitrosalicylic acid (DNS) [16]. To 1 mL of the test sample, 1 mL of DNS reagent was added and mixed thoroughly. The resulting mixture was then heated in boiling water for 5 min. After the mixture cooled to room temperature, its absorbance at 535 nm was measured. The content of monosaccharides was expressed in g of glucose or fructose equivalent per 1 L of juice tested.

### 2.9. Identification and Quantification of Polyphenolic Compounds by the LC-MS-PDA–Q/TOF and UPLC-PDA Methods

The analysis of polyphenolic compounds was performed according to Wojdyło and Nowicka [17]. Analysis of polyphenolic compounds in grapevines hybrids fruit was carried out using an Acquity UPLC system (Waters Corp., Milford, MA, USA) equipped with a PDA and FL detector with mass detector G2 Q/TOF mass spectrometer (Waters, Manchester, UK). The determination of the phenolic components was carried out via the retention time (Rt) and the accurate molecular masses both at negative and positive ion mode. The anthocyanins were read at 520 nm, flavonols at 360 nm, flavan-3-ols at 280 nm, and phenolic acids at 320 nm. All samples were measured in triplicate, and the results were expressed as mg per 1 kg of fresh mass.

### 2.10. Statistical Analysis

Data were subjected to one-way and two-way analysis of variance and mean values were compared by Tukey’s test (*p* < 0.05). Statistical analysis was performed using Stat-graphics Centurion 19 (Statgraphics Technologies, Inc., The Plains, VA, USA) statistical software.

## 3. Results and Discussion

Different varieties of *Vitis hybrid* vines are plants that are increasingly grown by farmers and growers around the world [18]. At the same time, consumers’ attention to the health effects of consumed food is increasing. Studies conducted so far revealed *Vitis* grape fruits (of many species and varieties) as those parts of the grapevine that have a high capacity to synthesize valuable secondary, and for this reason, the relevant parts of *Vitis* grape fruit are considered as a raw material with therapeutic and preventive effects against civilization diseases, such as cardiovascular disease (CVD) and cancer [19].

Research showed that resveratrol, present in high concentrations in grape skin (mainly red varieties) and red wine, exerts a number of beneficial health effects also within the central nervous system. It has been confirmed that resveratrol increases endogenous cellular antioxidant defenses, thus exerting anti-inflammatory (incl. by lowering the concentration of C-reactive protein in the blood) and anti-apoptotic effects against central nervous system cells (incl. by stimulating kinase C and reducing the toxicity of β-amyloid in brain neurons) [20].

Studies conducted to date report that consumption of *Vitis* hybrid grape fruit supports metabolic balance by improving glucose tolerance and pancreatic lipase inhibition, induced by proanthocyanidins containing flavan-3-ol, catechin and epicatechin molecules. This, in turn, has been implicated in reducing the risk of type II diabetes, inflammation and obesity. It is indicated that consumption of *Vitis* grape fruit is associated with increased expression of glucose transporter type 4 (GLUT4), resulting in improved conditions for glucose transport from the bloodstream. Furthermore, that antioxidant substances contained in grapes may be agonists of peroxisome proliferator-activated receptors-γ (PPAR-γ), thus showing anti-inflammatory effects [21].

Oxidative stress and associated damage to the glucose transporter i.e., glucose transporter type 4 (GLUT4) and peroxisome proliferator-activated receptor-γ (PPAR-γ) underlie the mechanism of diabetes and obesity development. So, it can be assumed that foods of particular health-promoting value are those groups of grapes that, in addition to possessing the property of acting on glucose transporter type 4 (GLUT4) and peroxisome proliferator-activated receptors-γ (PPAR-γ), also have high antioxidant properties, (providing oxidative protection to these receptors) [22].

In studies conducted so far, it has been shown that fruits of interspecific hybrids grown in Poland contain phenolic compounds belonging to both multiple groups, such as flavonoids (anthocyanins, flavonols, flavan-3-ols), proanthocyanidin tannins, phenolic acids and stilbenoids [23]. It is worth noting that only a few key reports on *Vitis* hybrid grapevines have been published so far, focusing mainly on the nutrient, micro and macronutrient requirements of these plants, on the description of fruit physicochemical characteristics, or on the process of creating new genotypes of these plants.

Therefore, we have characterized the bioactive properties of *Vitis* hybrid grapevine fruits grown in Poland in a temperate warm transitional climate (typical of temperate latitudes) and attempted to answer the question of whether, in terms of the content of phenolic compounds and antioxidant activity, hybrid grapevine varieties grown in Poland, are comparable with other grapevine varieties grown in other regions of the world.

### 3.1. Antioxidant and Oxidoreductive Activities of the Hybrid vines Fruit Fraction Studied

The antioxidative activity expressed as radical scavenging and oxidoreductive activities of skins, fruit pulp and juice of the tested Vitis hybrid grape cultivars grown in the Rzeszow Sub-Hills (in the Lower San Valley) in Poland is presented in Table 2.

#### 3.1.1. DPPH Assay

The average antioxidant activity of grape skin ranged from 3.31 to 4.34 (mg TE for 1 g of r.m.) for the cultivars Alwood and Beta, respectively, while the pulp ranged from 1.59 mg TE (for 1 g of r.m.) for V68021, to 1.89 mg TE for Michigan. The highest antioxidant activity (AA) in the skins (measured by the DPPH method) was found for the Beta variety (4.34 mg TE/g f.m.). These results correspond with the study of Costa and Ferreira (2021) [24], who reported that fruits of the BRS Cora cultivar (which are similar in color at maturity to Beta) exhibit AA ranging from 3.6 to 4.64 mg TE/g f.m. We also found the highest AA value for fresh fruit pulp of white Michigan (1.89 mg TE/g f.m.), while for Beta (black grapes), we obtained a value of 1.77 mg TE/g f.m. More diverse results were reported by Yilmaz et al. (2015) [25] for the pulp of the white variety 53/1 obtained 17.63 mg TE/g f.m., while for the variety Alphonse (black) reported 1.67 mg TE/1 g f.m.

The antioxidant activity of the juice ranged from 1.90 mg TE g (in 1 mL) for Beta, to 2.06 mg TE g (in 1 mL) for Alwood and Minnesota. The results indicated that there were no statistically significant differences in antiradical activity vs. DPPH between juice and pulp for all grape varieties tested, while the antiradical activities of skins were significantly different from the other fractions. However, there were no significant differences in skin’s antioxidant activity between the cultivars Michigan, Minnesota, V 68021 and Beta.

Our results also showed that there were no significant differences in antioxidant activity between juice and pulp for all the grape varieties studied, while the AA of the skin significantly exceeds the values of the other fractions—juice and pulp.

**Table 2:** Antioxidant activity and oxidoreductive potential of the fruits of 5 Vitis hybrid grape cultivars grown in the Rzeszów foothills (in the Lower San Valley) in Poland.

Fraction		Alwood	Beta	Michigan	Minnesota	V 68021	Variety	Fraction	Variety × Fraction
DPPH [TE]									
skins	[mg/g f.m.]	3.31 ± 0.06 ^a^	4.34 ± 0.06 ^d^	4.18 ± 0.02 ^cd^	3.99 ± 0.10 ^c^	3.60 ± 0.13 ^b^	ns	***	ns
pulp		1.80 ± 0.03 ^bc^	1.77 ± 0.03 ^b^	1.89 ± 0.01 ^c^	1.84 ± 0.01 ^bc^	1.59 ± 0.08 ^a^			
juice	[mg/mL]	2.06 ± 0.51 ^a^	1.90 ± 0.45 ^a^	1.98 ± 0.48 ^a^	2.06 ± 0.42 ^a^	1.92 ± 0.45 ^a^			
ABTS [TE]									
skins	[mg/g f.m.]	2.36 ± 0.05 ^a^	6.60 ± 0.12 ^d^	5.73 ± 0.09 ^c^	5.60 ± 0.13 ^c^	4.68 ± 0.12 ^b^	***	***	***
pulp		0.42 ± 0.00 ^b^	1.05 ± 0.01 ^d^	0.58 ± 0.01 ^c^	0.59 ± 0.01 ^c^	0.34 ± 0.01 ^a^			
juice	[mg/mL]	2.46 ± 0.02 ^a^	2.47 ± 0.03 ^a^	2.50 ± 0.01 ^a^	2.48 ± 0.03 ^a^	2.45 ± 0.05 ^a^			
FRAP [FeSO_4_^−2^]									
skins	[mg/g f.m.]	4.08 ± 0.15 ^d^	2.33 ± 0.23 ^a^	2.92 ± 0.23 ^bc^	2.85 ± 0.21 ^b^	3.42 ± 0.16 ^c^	***	***	***
pulp		0.07 ± 0.01 ^a^	0.17 ± 0.02 ^b^	0.22 ± 0.00 ^c^	0.09 ± 0.01 ^a^	0.10 ± 0.01 ^a^			
juice	[mg/mL]	0.13 ± 0.00 ^a^	0.16 ± 0.01 ^b^	0.15 ± 0.01 ^ab^	0.13 ± 0.01 ^a^	0.16 ± 0.01 ^b^			
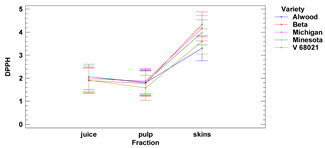					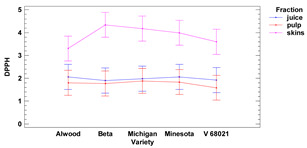				
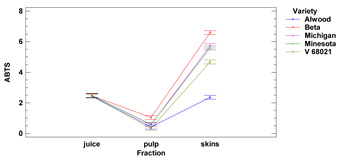					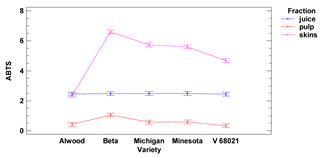				
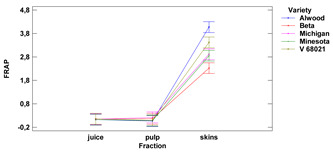					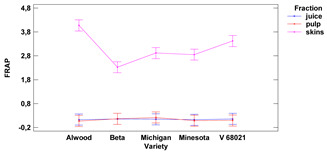				

Lower-case letters mean values significantly different at *p* ≤ 0.05 in the rows. Asterisks represent the second order interactions analysis, ***—significantly different at *p* ≤ 0.001, ns—not significantly different, f.m.—fresh mass. TE—Trolox equivalent. Two-way ANOVA charts of factors (variety and fraction) interactions are given below for each assay type.

The results of Baiano (2020) [26] do not confirm the observed trend, as more than 4.5 times higher AA was recorded in the pulp of the Red Globe cultivar than in the skins, however, the extraction of bioactive substances was conducted from the skins, without their prior crushing, which significantly affects the level of extracted compounds. In contrast, similar to ours, observations were made by Kupe et al. (2021) [27], who, for Karaerik dark grape skins, found antioxidant activity at levels of 10.80–13.40 μmol TE/g f.m., while for pulp at levels of 2.76–3.47 μmol TE/g f.m., and AA was more than 3–4 times higher for skins comparing with pulp.

Lingua et al. on the other hand, determined the AA value (based on the sample’s ability to protect the survival of *Saccharomyces cerevisiae* exposed to H_2_O_2_) for whole Syrah grapes, which ranged from 14% to 20%, while for the skin it ranged from 8% to 16%. The observed predominance of the whole grape extract is probably due to the fact that the bioactive compounds also included substances contained in the seeds, which increased the bioactive potential of the whole fruit extract compared to the exclusive skin extract. The lowest AA was described for Syrah fruit wine, where an increase in AA ranging from 9% to 15% was observed [28]. However, in this case, it is difficult to separate the bioactive effects from those of ethanol.

#### 3.1.2. ABTS Assay

The average antioxidant activity of grape skins measured by the ABTS method ranged from 2.36 to 6.60 mg TE (for 1 g of r.m.) for the Alwood and Beta, respectively. The antioxidant activity vs. ABTS for pulp ranged from 0.34 mg TE for V68021 to 1.05 mg TE (for 1 g of r.m.) Beta, while of the juice ranged from 2.45 TE g (in 1 mL) to 2.50 TE g (in 1 mL) for V68021 and Michigan, respectively. There were statistically significant differences in antioxidant activity vs. ABTS between juice and pulp for all grape varieties tested. In addition, there were significant differences in the antioxidant activity of skins compared to pulp (for Michigan, Minnesota, V68021 and Beta) and skins compared to juice (for all 5 grapevine cultivars tested). There were no significant differences observed in the antiradical activity of skins and pulp for the cultivar Alwood.

Using an ABTS assay, we observed that AA for the Michigan (white) variety skins was at 5.73 mg TE/g f.m. while for Beta (black) it was at 6.6 mg TE/g f.m. These values were several times higher than the values obtained by Yilmaz et al. (2015) [21] for the white varieties 85/1 (1.19 mg TE/g f.m.) or 86/1 (1.2 mg TE/g f.m.) and, on the other hand, several times lower for black varieties Isabella (1.19 mg TE/g f.m.) and 91/3 (1.21 mg TE/g f.m.). In pulp for the Michigan variety, the AA was at 0.58 mg TE/g f.m., while for the dark pulp of the Beta variety, we observed the AA at 1.05 mg TE/g f.m. Yilmaz et al. [25] reported for pulps of grapevine 85/1 and 86/1 (light varieties) 0.21 mg TE/g f.m. and 0.26 mg TE/g f.m., respectively, while of the pulp for Isabella grapevine it was 0.43 mg TE/g f.m. The higher AA values we obtained may be correlated with antioxidant groups that react strongly with the ABTS cation radical, such as phenolic glycosides. It is possible that the high content of these compounds caused rapid decomposition of the ABTS radical cations, as it has been confirmed that one molecule of phenolic glycoside can react with three molecules of ABTS radical cation [29].

The clear differences were observed between AAs for each fruit fraction, regardless of the cultivar (except for the cultivar Alwood, whose juice and skin AAs were not significantly different from each other). The lowest AA results were obtained for pulp, while skins showed the highest results. Our results are similar to those obtained by Kupe et al. (2021) [27], where for black grape skins Karaerik (a cultivar grown in Turkey) AA was found to be 3.10–4.14 μmol TE/g f.m., while for pulp, AA was found to be on average 5–6 times lower (0.50–0.98 μmol TE/g f.m.).

#### 3.1.3. FRAP Assay

The average reducing activity (RA) of grape skin ranged from 2.33 to 4.08 mg FeSO_4_^−2^ (for 1 g of f.m.) for the cultivars Beta and Alwood, respectively, for the pulp ranged from 0.07 mg FeSO_4_^−2^ (for 1 g of r.m.) for Alwood to 0.22 mg FeSO_4_^−2^ (for 1 g of f.m.) for Michigan. The reducing activity of the juice ranged from 0.13 mg/mL for Alwood and Minnesota to 0.16 mg/mL FeSO_4_^−2^ for the V 68021. There were no significant differences between juice and pulp for all five grape varieties tested. The reducing activity of skins was significantly different from juice and pulp (the highest difference was found for the Alwood variety). Furthermore, there were no significant differences in the reducing activity (of both juice and pulp) between the five studied varieties.

Similar results to ours were obtained by Kupe et al. [27], where Karaerik black grape skins revealed RA of 35.44–46.10 μmol TE/g f.m., while pulp was found to be on average more than 40 times lower, at 0.7–1.28 μmol TE/100 g f.m. Interestingly, there were no significant differences in RA (both juice and pulp) between the different grape varieties. Probably the high RA of grape skins is related to the high ability of the fruit to accumulate phenolic compounds in the skin, as well as ascorbic acid and β-carotene [30].

### 3.2. Polyphenolics, Flavonoids and Anthocyanins Content of the Hybrid Vines Fruit Fraction Studied

#### 3.2.1. Total Polyphenolic Compounds Content (TPC)

The content of polyphenolic compounds, flavonoids and anthocyanins in skins, fruit pulp and juice of the tested *Vitis* hybrid grape cultivars grown in the Rzeszow Sub-Hills (in the Lower San Valley) in Poland is presented in Table 3. The values of total polyphenolic compounds (TPC) per 1 g in skins ranged from 2.58 to 16.33 mg GAE for Beta and Alwood, respectively, while for pulp ranged from 0.02 to 0.37 mg GAE for Beta and Michigan, respectively. The values of total polyphenolic compounds in fresh juice ranged from 0.02 to 1.30 mg GAE/1 mL for Alwood and Michigan, respectively. There were no (except Beta and Michigan) significant differences between the TPC in juice and pulp. It was observed that the total content of phenolic compounds in skins for all cultivars was significantly different compared to the values of phenolic compounds in juice and pulp (the highest difference was found between skins and juice and between skins and pulp of Alwood cultivar).

The highest content of phenolic compounds (TPC) was observed for Alwood skins at 16.33 mg GAE/g f.m. Compared to the TPC for dark varieties Isabel (1.51 mg GAE/g f.m.), Hamburg Misket (1.43 mg GAE/g f.m.) or Alphonse (1.46 mg GAE/g f.m.) obtained by Yilmaz et al. (2015) [25] the TPC values obtained by us were more than 10 times higher.

The Alwood variety we studied (as a result of heterozygous breeding) is characterized by enhanced traits, which are manifested by increased biosynthesis of polyphenolic compounds). According to the assumption proposed by Donovan, genotypes of hybrid varieties may contain beneficial alleles from the initial parental species, which result in greater environmental adaptation and thus a higher chance of survival [31].

There were no (except for Beta and Michigan cultivars) significant differences between TPC values in juice and fruit pulp for Alwood, Minnesota and V 68021 cultivars. However, we observed that TPC in skins for five tested grapevines, differed significantly between juice and pulp. Similar results were obtained by Kupe et al. (2021) [27], where for Karaerik black grape skins, TPC was found to be 111–149 mg GAE/100 g f.m., while for pulp, polyphenol content was found to be 2.77–3.715 mg GAE/100 g f.m. Partial confirmation of the trend we observed are the results obtained by Lingua et al. for very similar (Syrah variety) or significantly higher (Merlot variety) TPC value in skins (2122 mg GAE/g d.m.) compared to whole grape fruit (1458 mg GAE/g d.m.). At the same time, the quoted results show that there are no TPC differences between the fraction of whole fruits (1458 mg GAE/g d.m.) and the obtained wine from these fruits (1475 mg GAE/L) for the Merlot variety [28].

We observed no differences (except for Michigan) in TPC between the fruit pulp fraction and the grape juice. All the skins revealed high TPC, suggesting the possibility of using this raw material as a source of bioactive compounds with high antioxidant and antimicrobial activity, so they could find great applications in the food, pharmaceutical and cosmetic industries [32]. Baiano (2020) [26] also showed that TPC in grape (Red Globe variety) skins (1.32 mg GAE/g f.m.) was almost four times higher than polyphenolic content in fruit pulp (0.35 mg GAE/g f.m.).

In our study, the highest value of TPC in skins was characterized by the Alwood cultivar, which could be due to the high content of anthocyanins. Studies on the properties of polyphenolic compounds from grape pomace indicate their strong antiproliferative and proapoptotic effects against cells of the HaCaT cell line, which is a commonly used model of human keratinocytes (primary keratinocytes). Therefore, it is indicated that grape skins can be a valuable raw material used to obtain preparations with cytostatic (anticancer) properties [33].

#### 3.2.2. Total Flavonoid Content (TFC)

The total flavonoid content of the skins showed average values ranging from 113.2 to 269.8 μg QE/1 g of r.m. for the cultivars Michigan and Beta, respectively. Values of total flavonoid content in fresh fruit pulp ranged from 133.6 to 311.2 μg QE/1 g of r.m. for the cultivars Michigan and Alwood, respectively. The values of total flavonoid content in fresh juice ranged from 7.60 to 55.10 μg QE/1 mL for Michigan and Beta, respectively. The flavonoid content (TFC) of skins and pulp differed significantly from juice. In addition, we observed that there were no statistically significant differences in TFC between skins and pulp for Beta, Michigan and Minnesota cultivars nor in juice between all five grape varieties studied. Our results are consistent with those obtained by Kupe et al. [27], where for Karaerik grape skins, TFC was found to be 0.9–2.4 mg quercetin/100 g f.m., while for pulp TFC was found to be on average 5–6 times lower, at 0.1–0.2 mg quercetin/100 g f.m.

Lingua et al. [28] observed partially similar trends for pulp and skins from red grape varieties Syrah, Merlot and Cabernet Sauvignon. They observed that the content of selected flavonoids (e.g., quercetin) in the skins (251.1 mg/kg d.m.) is 100 to more than 230 times higher than in the whole fruit (2.3 mg/kg d.m.) [29]. However, in the cited study, quercetin content was not analyzed in the fruit pulp itself, but in the whole fruit. This may have resulted in an overestimation of the total flavonoid content, as homogenized plant material included both skins, pulp and seeds of the grapes [28]. In contrast, grape seeds are seen as a part of the grape fruit that is highly abundant in bioactive compounds [34]. Studies indicate that grape seeds may contain a similar amount of flavonoids (7.61 g catechins/100 g d.m.) as in skins (7.76 g catechins/100 g d.m.) [35]. The high flavonoid content of grape skins is indicated by the results obtained by Pfukwa et al. (2019) [35], who confirmed that pomace of the Pinotage variety contains this group of compounds at 7.76 g/100 g d.m. (in catechin equivalent).

#### 3.2.3. Total Anthocyanin Content (TAN)

Values of total anthocyanin content (TAN) in skins ranged from 0.33 to 2.38 mg DE (for 1 g) for the Michigan and Alwood, respectively, while for pulp ranged from 0.04 mg DE for the cultivar Michigan to 0.55 mg DE/1 g for the cultivars Alwood and Beta. TAN content in fresh juice ranged from 0.015 mg to 0.185 mg DE/1 mL for the Michigan and Beta, respectively. The TAN content between skins and juice and between skin and pulp, were significantly different for all five grape varieties tested. Total anthocyanin contents between pulp and juice were found to be significantly different for the four grape cultivars tested—Minnesota, V 68021, Beta and Alwood. There were no statistically significant differences in anthocyanin content in juice between the grape varieties.

The total content of anthocyanins (TAN) in our study for skins was significantly higher than in pulp and juice (for all five grape varieties studied). Recalculating on delphinidin equivalent alone, Lingua et al. obtained a higher content of this anthocyanin in the skins of the varieties studied (43.9 mg/kg f.m. for Syrah) than in the whole fruit (8.0 mg/kg f.m.), which corresponds with our observations [28,29].

However, also Lingua et al. (2016) [28] obtained a lower TAN in skins for the Syrah cultivar (142.2 mg/kg d.m. than in whole fruit (380.5 mg/kg d.m.) using malvidin-3-glucoside.

Partial confirmation of our results is provided by the results obtained by Lingua et al., where it was found that the TAN (in malvidin-3-glucoside equivalent) in grape skins is 1.6 to 2 times higher than the anthocyanin content in wine extracted from grapes of the analyzed varieties (such as Syrah, Merlot and Cabernet Sauvignon). However, in terms of delphinidin content alone for the Syrah variety, Lingua et al. obtained a very low (below the detection threshold) content of this anthocyanin in wine compared to the delphinidin content in the skins (43.9 mg/kg d.m.) [28]. This suggests that during wine-making, only partial leaching of some anthocyanin groups from the skin occurs at the maceration process stage, and the vast majority of them may still remain in the grape skin.

This observation is very much in line with our results, where in the juice for the Alwood variety we recorded a TAN of 0.115 mg/mL, while in the skin it was 2.38 mg/g f.m. We found that for the grape varieties tested (except the Michigan variety), the TAN in the pulp (e.g., 0.55 mg/mL for Beta) is significantly higher than the anthocyanin content in the juice (0.185 mg/mL for Beta). An analogy to this study can be found in the results of Lingua et al., where a significantly lower anthocyanin content was observed in Merlot wine (46.6 mg/L) than in whole fruit (251.5 mg/kg d.m.) [28]. In addition, Pfukwa (2019) [35] reported the same trend and indicated that the skins of the Pinotage variety have an anthocyanin content as high as 0.173 g cyanidin-3-glycoside/100 g d.m. 

### 3.3. Total Content of Reducing Sugars (GLUE and FRUE)

Sugar concentration values (expressed in glucose equivalent) ranged from 7 to 38.9 g Glu/L and expressed in fructose equivalent ranged from 6.28 to 41.49 g Fru/L for Alwood and Michigan, respectively. The sugar content in fruit juice of *Vitis* hybrid grapevines cultivated in the Rzeszow Foothills (in the Lower San Valley) in Poland is presented in Table 4.

Baiano analyzed the juices of hybrid vines grape juices and commercially available grape juices obtaining values from 168 to 173 g/L for white variety Italia spp (*V. vinifera*) and 147–151 g/L Red Globe spp (*V. vinifera*), confirming the trend of higher sweetness in white varieties [26]. Barros et al. also verified the content of sugars (especially glucose and fructose) in the juice of two varieties of *V. labrusca* and, despite reporting the strong relation with location and year of harvest, observed the average glucose content of 39.7–62.47 g/L for Bordo and 54.05–72.16 g/L for Concord cultivar, respectively. Fructose content was higher than glucose and was reported as 48.12–80.04 g/L for Bordo and 57.03–78.46 for the Concord variety, respectively. The sugar content was high compared to results obtained by us, but it was probably connected with the climate and the varieties/cultivars developed in the country of origin. As Barros et al. stated, those varieties are of high importance to Brazil as these and their hybrid cultivars represent more than 80% of the volume of grapes processed in this country [36]. So they were previously selected to comply with the market requirements. Polish cultivars which we studied are an amateur cultivation for decorative and personal use, but all of them revealed lower simple sugar content, especially Alwood (7.0 g/L GLUE and 6.28± g/L FRUE) and Beta (16.7 g/L GLUE and 13.18 g/L FRUE) cultivars (black varieties) which was simultaneously abundant in anthocyanins. Such a profile of bioactive compounds can indicate the high utility of the Alwood cultivar for direct consumption.

### 3.4. Analysis of Phenolic Compounds by Thin-Layer Chromatography Coupled with Mass Spectrometry (LC-MS-PDA)

The analysis carried out by LC/MS-Q/TOF (Table 5) indicated the content of total 34 compounds that form the polyphenols profile of the grapevines hybrids. These compounds can be classified into five different fractions, i.e., flavan-3-ols, anthocyanins, flavonols, phenolic acids, and non-flavonoid stilbenes.

The results of the research carried out with the LC-MS-PDA–Q/TOF and UPLC-PDA methods confirmed that anthocyanins and flavan-3-ols mostly shaped the final concentration of polyphenolic compounds. The remaining compounds, i.e., phenolic acids and flavonols, constituted about 20% of the total polyphenols. In turn, considering the fruit’s individual fractions—juice, pulp and skin, it was shown that the most abundant source of bioactive compounds were the skins, and then the pulps and juices. Interestingly, it was shown that the cultivars containing anthocyanins in the composition of the skins had a higher concentration of polyphenolic compounds in the juice, while in the cultivars where the presence of anthocyanins was not observed, the juice was less abundant with these compounds. It proves the high migration of polyphenolic compounds from the skin during the pressing process and what has been shown earlier that the anthocyanin content in the grape largely influences the final concentration of the total content of polyphenols.

A total of 13 anthocyanins were detected in all analyzed grapes, including 3 delphinidins, 6 malvidins, 2 peonidins, and 2 petunidins (Table 5). Anthocyanins were present in acylated form, but also in combination with *p*-coumaric and caffeic acids. The profile of anthocyanins differed between the cultivars, and only in three of them were these compounds (Minnesota, Beta, and Alwood) detected.

The source of this fraction of compounds was the skins, as they accumulate red pigments. Hence, as a result of the pressing process, these compounds also appeared in the juice, but only in the Beta and Alwood cultivars, which results directly from the initial concentration of these compounds in the fruit. Others authors confirmed that in the vast majority of red, grape cultivars, anthocyanins are synthesized only in the skin, and are just only few cultivars where a typical in that they accumulate anthocyanins in the flesh as well [37]. The content of these compounds is determined mainly by the genetics of cultivar [23].

Dephinidin-3-O-rutinoside ([M+H]+ at m/z = 611), delphinidin-3-O-rhamnoside ([M+H]+ at m/z = 449), and peonidin-3-O-glucoside ([M+H]+ at m/z = 463) were identified in Minnesota, Beta and Alwood skins. Malvidin-3-O-glucoside ([M+H]+ at m/z = 493), malvidin-3,5-di-O-glucoside ([M+H]+ at m/z = 655), and acylated forms of anthocyanins-delphinidin-3-O-(6′′-acetyl)-glucoside, malvidin-3-O-(6″-acetyl)-glucoside were identified just only in Beta cv., while the anthocyanin’s combination with phenolic acids (malvidin-3-O-(6-O-caffeoyl)-glucoside; petunidin-3-O-(6″-p-coumaroyl)-glucoside; malvidin-3-O-(6″-p-coumaroyl)-glucoside) were characteristic both for Alwood, and Beta cvs.

The content of anthocyanins ranged from 11.15 mg/kg (Minnesota skin) to 716.57, and 728.83 (Beta, and Alwood skin, respectively), while it should be noted that in the other tested cultivars, no anthocyanin content was detected. Peonidin-3-glucoside accounted for over 60% of anthocyanin content in these cultivars, around 20% was dephinidin-3-O-rutinoside, rest of the detected compounds occurred in much smaller amounts. A similar share of compounds was noted by Torchio et al. in Avana and Nebbiolo cultivars [38].

In all the tested cultivars, the content of flavan-3-ols was the highest in the skin, then in the pulp, and the juices were the least rich in bioactive compounds. While anthocyanins easily migrate to the juice during the pressing process, flavan-3-ols are more difficult due to their structure and connections in the skins. Probably a better effect for the migration of polyphenolic compounds would be obtained at the time of intensive skin crushing or/and carrying out the depectinization process. The appropriate crushing before pressing causes damage to cell walls, and thus, the migration of bioactive fruit compounds to juice. In turn, pectolytic enzymes are applied to the degradation of cell walls, which enables the degradation of the tissue structure by breaking pectins. This process allows for an additional increase in pressing yield and, consequently, in the flawan-3-ols content of juice [39,40].

The total content of flavan-3-ols in skins ranged from 37.62 mg/kg (Michigan cv.) to 205 mg/kg (Alwood cv.), in the case of pulp from 16.48 mg/kg (Michigan cv.) to 85.73 mg/kg (V68021 cv.), and in juices from 8.82 mg/kg (Michigan cv.) to 47.15 mg/kg (Beta cv.). These values were mainly shaped by (+)-catechin and (−)-epicatechine ([M-H]−at m/z 289.00), which were present in almost all analyzed samples. The (+)-catechin was not determined only in Michigan’s pulp and Alwood’s juice, while the (−)-epicatechin was not in Minnesota’s pulp. Three procyanidin dimers have also been identified in grape fruit ([M-H]−at m/z 578, Rt—2.819; 3.625; 4.392 min). Other authors have also detected this composition in other cultivars of grapes [23,41].

The total content of polyphenols ranged from 9.16 mg/kg in the case of Michigan pulp to 1016.52 mg/kg in Alwood skin (Table 6).

The content of phenolic acids (Table 6) ranged from 0.45 mg/kg (Minnesota juice) to 43.87 mg/kg (Alwood skin). The most common acid was caftaric acid ([M-H]—at m/z 311), detected in almost all examined skins, pulps, and juices (expect Minnesota juice and skin). Interestingly, some compounds were marked only in white and pink grapes: gallic acid ([M-H]—at m/z 169), gentisic acid ([M-H] −at m/z 193), and caffeoyl glucose ([M-H]—at m/z 341). Chlorogenic acids were presented only in Beta’s skin and V68021’s pulp, while caffeic acid was detected in all tested cultivars.

The two analyzed cultivars also contained trans-resveratrol belonging to (E)-stilbene family, defined as phytoalexins. It was found in white and rose cultivars: Michigan (skin and pulp) and Minnesota (skin and pulp). The concentration of resveratrol was very low, and it ranged from 1.75 mg/kg (Michigan pulp) to 6.11 mg/kg (Minnesota skin). Many studies report low or undetectable levels of stilbenes. This compound is synthesized by plants in response to stress. Therefore, there are possibilities for improving resveratrol content in grapes by biotic and abiotic factors. However, it should be remembered that grapes grown at low temperatures (as in Poland) contain fewer stilbenes than those ripening in a warm climate [42,43].

The last group of bioactive compounds found in grapes was flavonols. In total, seven compounds of this fraction were identified; they were two derivatives of myricetin, three derivatives of quercetin, and two derivatives of isorhamnetin. These compounds were mainly identified in the skins, although in two cases, they migrated to juice during the pressing process-Michigan and Alwood cvs. The dominant compound in the presented cultivars was quercetin-3-O-rutinoside ([M-H]−at m/z 609). Rest of them were found in much smaller content. Michigan’s skin was characterized by the highest content of flavonols (64.48 mg/kg), after that was the skin of Alwood (38.81 mg/kg) >> Minnesota (11.15 mg/kg) >> V68021 (8.47 mg/kg), and Beta cv. where flavonols were not detected. Both the qualitative profile and the concentration of the discussed compounds are also confirmed by other authors [23,44]. They point out that flavonols are a class of flavonoid located in grape skins, and quercetin structures are the main flavonol derivatives accumulated there [44].

The results presented by us may indicate significant differences in the profile of bioactive compounds (mainly phenolic) of particular fractions of the examined hybrid grapevine fruits. Grape skins were found to be the most valuable fraction able to provide the greatest health benefits to the consumer. However, fruit pulp and juice of the studied hybrid grape varieties present similar bioactive properties and thus may be characterized by similar health-promoting effects on the organism of consumers consuming them.

## 4. Conclusions

As a result of analytical studies, it turned out that hybrid grapevine cultivars (Alwood, Beta, Michigan, Minnesota and V68021), grown in Poland, showed equally high total antioxidant activity compared to other hybrid cultivars, such as BRS Cora or Isabelle, but grown in warmer regions of southern Europe. This confirms the significant effect of a cool climate on increasing the accumulation of bioactive substances in the plant, which is related to the adaptability of Vitis species to different types of climate.

The abundance of polyphenolic bioactive compounds, mainly anthocyanins, flavan-3-ols and flavonols in the skins of the grapes studied, indicates that hybrid grape cultivars, so far grown in Poland mainly as dessert and garden plants, have great potential to become a highly valuable raw material in the food and pharmaceutical industries.

The obtained results indicate that hybrid grape varieties grown in Central and Eastern Europe can be a more valuable raw material for functional food production than traditional noble Vitis varieties. Dark hybrid grape varieties, such as dessert-growing Beta or dessert-growing Alwood, due to their strong antioxidant properties, are potential raw materials for the production of liqueurs or as an additive (in dried form) to bakery products, among others. On the other hand, light hybrid grape varieties, such as the Michigan processing-dessert variety, may be treated as healthy, low-calorie and tasty snacks, at the same time supplementing the diet with valuable bioactive substances, such as trans-resveratrol.

## Figures and Tables

**Table 1:** Morphocharacteristcs of grapes variety.

Michigan	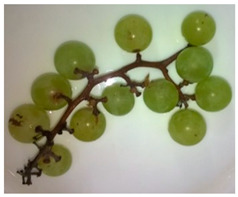	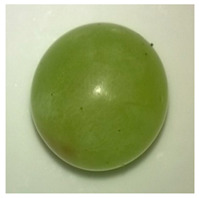
V 68021	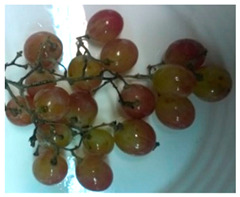	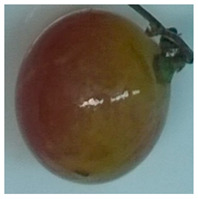
Minnesota	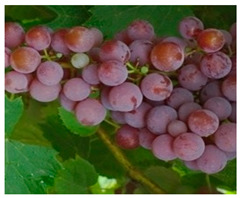	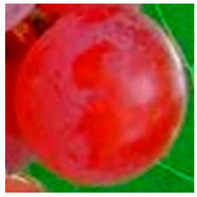
Beta	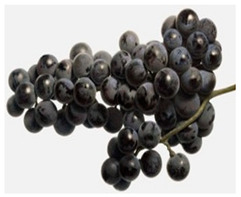	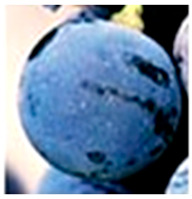
Alwood	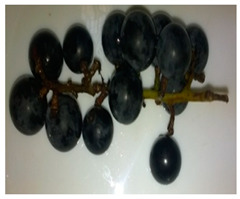	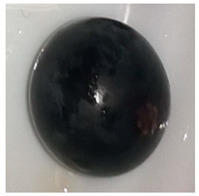

**Table 3:** Equivalents content for polyphenolic compounds, flavonoids and anthocyanins in skins, fruit pulp and juice of the studied Vitis hybrid grape cultivars grown in the Rzeszów Podgórze area (in the Lower San Valley) in Poland.

Fraction		Alwood	Beta	Michigan	Minnesota	V 68021	Variety	Fraction	Variety × Fraction
TPC [GAE]									
skins	[mg/g f.m.]	16.33 ± 0.08 ^e^	2.58 ± 0.16 ^a^	6.37 ± 0.13 ^b^	7.39 ± 0.10 ^c^	8.72 ± 0.18 ^d^	***	***	***
pulp		0.03 ± 0.01 ^a^	0.02 ± 0.03 ^a^	0.37 ± 0.01 ^c^	0.25 ± 0.05 ^b^	0.30 ± 0.01 ^b^			
juice	[mg/mL]	0.02 ± 0.02 ^a^	0.56 ± 0.10 ^b^	1.30 ± 0.09 ^bc^	0.27 ± 0.04 ^c^	0.48 ± 0.16 ^d^			
TFC [QE]									
skins	[µg/g f.m.]	251.0 ± 10.0 ^c^	269.8 ± 12.9 ^c^	113.2 ± 8.9 ^a^	172.9 ± 9.8 ^b^	126.2 ± 11.5 ^a^	***	***	***
pulp		311.2 ± 15.0 ^c^	310.7 ± 10.9 ^c^	133.6 ± 11.1 ^a^	196.1 ± 9.1 ^b^	179.0 ± 12.9 ^b^			
juice	[µg/mL]	34.50 ± 1.84 ^b^	55.10 ± 2.40 ^c^	7.60 ± 1.13 ^a^	30.70 ± 0.99 ^b^	31.90 ± 0.99 ^b^			
TAN [DE]									
skins	[mg/g f.m.]	2.38 ± 0.03 ^e^	2.13 ± 0.04 ^d^	0.33 ± 0.02 ^a^	1.18 ± 0.03 ^c^	1.08 ± 0.03 ^b^	***	***	***
pulp		0.55 ± 0.03 ^c^	0.55 ± 0.03 ^c^	0.04 ± 0.03 ^a^	0.36 ± 0.03 ^b^	0.44 ± 0.04 ^b^			
juice	[mg/mL]	0.12 ± 0.01 ^c^	0.19 ± 0.01 ^d^	0.02 ± 0.01 ^a^	0.09 ± 0.01 ^b^	0.09 ± 0.01 ^b^			
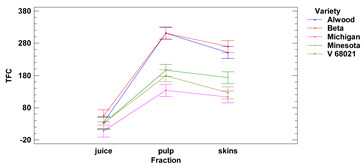					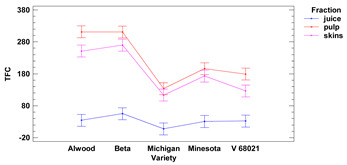				
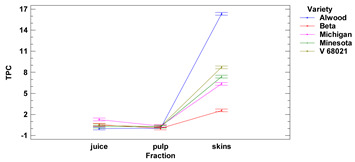					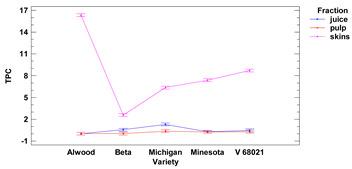				
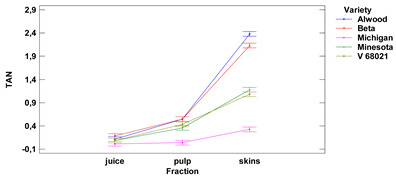					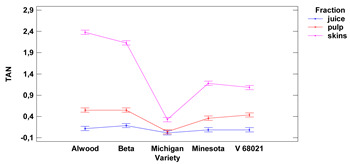				

Lower-case letters mean values significantly different at *p* ≤ 0.05 in the rows. Asterisks represent the second order interactions analysis, ***—significantly different at *p* ≤ 0.001, f.m.—fresh mass. DE—delphinidin equivalent, QE—quercetine equivalent, GAE—gallic acid equivalent. Two-way ANOVA charts of factors (variety and fraction) interactions are given below for each assay type.

**Table 4:** The total content of reducing sugars in the juice of the tested Vitis hybrid grape cultivars grown in the Rzeszów Sub-County (in the Lower San Valley) in Poland.

	Glucose [GLUE]	Fructose [FRUE]
	[g/L]	
Alwood	7.0 ± 0.5 ^a^	6.28 ± 0.39 ^a^
Beta	16.7 ± 0.7 ^b^	13.18 ± 0.59 ^b^
Michigan	38.9 ± 0.3 ^e^	41.49 ± 1.03 ^e^
Minnesota	26.0 ± 0.5 ^d^	26.86 ± 0.54 ^d^
V 68021	20.6 ± 1.0 ^c^	25.07 ± 0.54 ^c^

Lower-case letters mean values in the columns are significantly different at *p* ≤ 0.05.

**Table 5:** Phenolic compounds identified in grapevines hybrids flesh, skins, and juices by LC/MS-Q/TOF.

Compounds	Rt (min)	λmax (nm)	MS [M-H]^−^(m/z)	MS/MS [M-H]^−^(m/z)	Kind of Sample
**Anthocyanins**					
Dephinidin-3-O-rutinoside	4.193	522	611.14^+^	303.05	Minnesota—skin; Beta—skin, juice; Alwood—skin, juice
Malvidin-3,5-di-O-glucoside	4.444	522	655.27^+^	493.11/331.05	Beta—skin
Delphinidin-3-O-(6″-acetyl)-glucoside	4.724	520	507.11^+^	303.05	Beta—skin
Delphinidin-3-O-rhamnoside	4.750	522	449.02^+^	303.05	Minnesota—skin; Beta—skin, juice; Alwood—skin, juice
Malvidin-3-O-galactoside	5.281	527	493.13^+^	331.04	Beta—skin; Alwood—skin
Peonidin-3-O-glucoside	5.301	517	463.10^+^	301.07	Minnesota—skin; Beta—skin, juice; Alwood—skin, juice
Peonidin-3-O-rutinoside	5.433	522	609.63^+^	301.07	Beta—skin; Alwood—skin
Malvidin-3-O-glucoside	5.503	522	493.10^+^	331.04	Beta—skin
Petunidin-3-O-(6″-acetyl)-glucoside	5.885	525	521.12^+^	317.07	Beta—skin, juice; Alwood—skin
Malvidin-3-O-(6″-acetyl)-glucoside	6.971	529	535.23^+^	331.05	Beta—skin
Malvidin-3-O-(6-O-caffeoyl)-glucoside	7.320	531	655.17^+^	331.04	Beta—skin; Alwood—skin
Petunidin-3-O-(6″-p-coumaroyl)-glucoside	7.593	527	625.31^+^	317.07	Beta—skin; Alwood—skin, juice
Malvidin-3-O-(6″-p-coumaroyl)-glucoside	8.592	530	639.16^+^	331.05	Beta—skin; Alwood—skin
**Flavonols**					
Myricetin-3-O-glucoside	5.596	360	478.98	316.89	Minnesota—skin; Alwood—skin, juice
Myricetin-3-O-rhamnoside	6.251	349	464.36	316.89	Michigan—skin, juice
Quercetin -3-O-rutinoside	6.385	355	609.11	300.95	Minnesota—skin; Michigan—skin, juice; V68021—skin
Quercetin-3-O-glucuronide	6.553	354	476.95	300.95	Minnesota—skin; Michigan—skin; Alwood—skin, juice
Quercetin-3-O-glucoside	6.581	355	462.98	300.95	Michigan—skin; Alwood—skin, juice
Isorhamnetin-3-O-rutinoside	7.277	355	623.08	315.01	Michigan—skin; Alwood—skin
Isorhamnetin-3-O-glucoside	7.526	355	477.13	315.01	Michigan—skin; Alwood—skin, juice
**Phenolic acids and stilbenes**					
Gallic acid	1.386	272	169.11	124.98	Michigan—skin; Minnesota—pulp; V68021—pulp;
Gentisic acid	2.391	327	192.99	178.98/149.01	Michigan—pulp, juice;
Caftaric acid	3.160	323	311.04	178.98/149.01	Michigan—pulp, juice, skin; Minnesota—pulp; V68021—pulp, skin, juice; Beta—pulp, skin, juice; Alwood—pulp, skin, juice
Chlorogenic acid	3.818	325	353.21	191.05	V68021—pulp; Beta—skin
Caffeoyl glucose	4.089	328	341.01	178.98/160.98/135.00	Michigan—pulp, skin, juice; Minnesota—pulp, skin, juice; V68021—pulp;
Caffeic acid	4.103	323	179.07	135.00	Michigan—pulp, skin, juice; Minnesota—pulp, juice; V68021—pulp, juice; Beta—pulp, skin, juice; Alwood—pulp, skin
Coutaric acid	4.822	312	294.97	162.98	Michigan—pulp, skin, juice; V68021—juice; Beta—skin; Alwood—pulp, skin
Fertratic acid	5.318	327	325.02	192.99/149.01	Michigan—pulp, skin; V68021—pulp; Alwood—skin
Trans-resveratrol	7.153	304	227.00		Michigan—skin, juice; Minnesota—skin, juice;
**Flavan-3-ols**					
Procyanidin dimer	2.819	278	577.14	289.11	Michigan—skin; V68021—skin, pulp;
(+)-catechin	3.593	278	289.11		Michigan—skin, juice; Minnesota—pulp, skin, juice; V68021—pulp, skin, juice; Beta—pulp, skin, juice; Alwood—pulp, skin
Procyanidin dimer	3.625	279	578.13	289.11	Michigan—pulp, skin; Minnesota—pulp, skin; V68021—pulp, skin, juice; Alwood—pulp, skin
Procyanidin dimer	4.392	280	578.13	289.11	Michigan—skin, juice; V68021—juice; Beta—pulp, skin; Alwood—pulp
(−)-epicatechin	4.666	278	289.11		Michigan—pulp, skin, juice; Minnesota—skin, juice; V68021—pulp, skin, juice; Beta—pulp, skin, juice; Alwood—pulp, skin, juice

^+^[M + H]+ (m/z) for anthocyanins were obtained in the positive mode.

**Table 6:** The content of polyphenolic compounds in grapevines hybrids skin, juice and pulp.

Variety	Fruit Fraction	Anthocyanins [mg/1 kg]	Flavonols [mg/1 kg]	Phenolic Acids and Stilbenes [mg/1 kg]	Flavan-3ols [mg/1 kg]	Total Polyphenols [mg/1 kg]
Michigan	skin	nd	64.48 ± 6.13 ^a^	33.96 ± 3.75 ^b^	37.62 ± 0.48 ^g^	136.06 ± 10.36 ^d^
	juice	nd	8.37 ± 0.02 ^d^	2.56 ± 0.07 ^h^	8.82 ± 0.04 ^k^	19.75 ± 0.13 ^j^
	pulp	nd	nd	2.68 ± 0.06 ^h^	16.48 ± 0.98 ^j^	19.16 ± 1.04 ^j^
Minnesota	skin	11.15 ± 2.42 ^bc^	15.69 ± 1.01 ^c^	21.50 ± 0.93 ^c^	125.02 ± 8.52 ^c^	173.36 ± 12.88 ^c^
	juice	nd	nd	0.45 ± 0.09 ^k^	22.27 ± 0.10 ^i^	22.72 ± 0.19 ^i^
	pulp	nd	nd	2.27 ± 0.00 ^i^	40.75 ± 1.07 ^f^	43.02 ± 1.07 ^g^
V 68021	skin	nd	8.47 ± 0.38 ^d^	16.89 ± 1.12 ^d^	158.81 ± 6.27 ^b^	184.18 ± 7.77 ^c^
	juice	nd	nd	2.56 ± 0.24 ^h^	27.41 ± 1.33 ^h^	29.97 ± 1.57 ^h^
	pulp	nd	nd	3.38 ± 0.61 ^g^	85.73 ± 1.41 ^d^	89.11 ± 2.02 ^e^
Beta	skin	716.57 ± 11.03 ^a^	nd	11.21 ± 0.61 ^e^	86.83 ± 4.15 ^d^	814.61 ± 15.79 ^b^
	juice	8.51 ± 1.02 ^c^	nd	5.35 ± 0.46 ^f^	47.15 ± 4.10 ^e^	61.01 ± 5.59 ^e^
	pulp	nd	nd	5.43 ± 0.73 ^f^	46.61 ± 0.75 ^e^	52.04 ± 1.48 ^f^
Alwood	skin	728.83 ± 9.21 ^a^	38.81 ± 1.32 ^b^	43.87 ± 1.96 ^a^	205.00 ± 11.11 ^a^	1016.52 ± 23.60 ^a^
	juice	14.47 ± 0.32 ^b^	4.92 ± 0.03 ^e^	4.49 ± 0.79 ^f^	25.15 ± 2.11 ^h^	49.03 ± 3.25 ^fg^
	pulp	nd	nd	2.09 ± 0.04 ^j^	41.49 ± 1.91 ^f^	43.58 ± 1.95 ^g^

nd—not detected. Lower-case letters mean values in the columns significantly different at *p* ≤ 0.05.

## Data Availability

The data presented in this study are available on request from the corresponding author.
